# A simple, rapid, resin-free platform for purification of vascular endothelial growth factor using a calcium-responsive fusion protein

**DOI:** 10.1186/s40643-026-01063-y

**Published:** 2026-05-04

**Authors:** Jonghwan Lee, Heesun Park, KyuHyuk Im, Sanguine Byun, Sunghyun Kim

**Affiliations:** 1 Center for Convergence Bioceramic Materials, Korea Institute of Ceramic Engineering and Technology, 202 Osongsaengmyeong 1-ro, Cheongjusi, Chungcheongbuk-do 28160 South Korea; 2 Department of Biotechnology, Yonsei University, Seoul, 03722 Republic of Korea

**Keywords:** Vascular endothelial growth factor, Purification, Fusion protein, Calsequestrin, Single-chain variable fragment, Non-chromatographic purification

## Abstract

**Background:**

Vascular endothelial growth factor (VEGF) is widely used in regenerative medicine and therapeutic research. However, the purification of recombinant VEGF largely relies on affinity chromatography, which requires expensive chromatographic columns, specialized equipment, and multistep processing. These column-based workflows increase operational complexity and cost, particularly for large-scale production. Therefore, the development of an alternative purification strategy to conventional chromatography-based purification for VEGF is needed.

**Findings:**

In this study, we developed a chromatography-free VEGF purification strategy using an anti-VEGF-scFv–calsequestrin (CSQ) fusion protein that enables calcium-dependent affinity precipitation. The fusion protein retained strong binding affinity for VEGF (*K*_d_ = 1.1 nM) while exhibiting rapid and reversible Ca^2^⁺-dependent polymerization. Upon CaCl₂ addition, the anti-VEGF-scFv-CSQ–VEGF complex rapidly formed aggregates, enabling efficient separation of VEGF from impurities. Using this strategy, VEGF was purified within 30 min with a purity of 94% and a yield of 93%. SEC-HPLC analysis confirmed a purity of 94.3%, and host cell protein contamination was reduced from 1.44 × 10^4^ ppm to 774 ppm. The fusion protein also maintained stable purification performance over five repeated cycles, with VEGF recovery consistently maintained above 85%.

**Conclusions:**

These findings demonstrate that the scFv-CSQ fusion protein enables rapid separation of VEGF through calcium-dependent polymerization. This column-free mechanism reduces operational cost and technical complexity, highlighting its potential as an alternative to conventional chromatography-based purification.

**Graphical abstract:**

**Supplementary Information:**

The online version contains supplementary material available at 10.1186/s40643-026-01063-y.

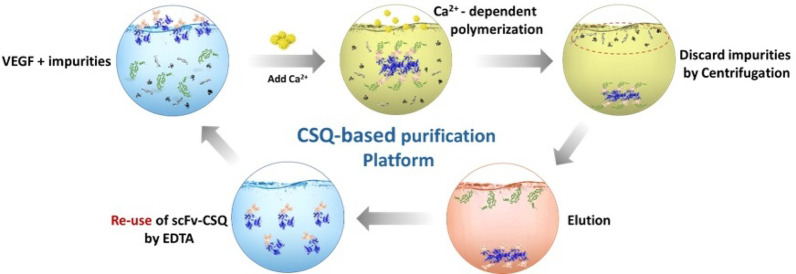

## Introduction

Vascular endothelial growth factor (VEGF) is a homodimeric glycoprotein that plays a critical role in angiogenesis by promoting endothelial cell survival, proliferation, and migration while increasing vascular permeability (Brown et al. 1997; Wiszniak and Schwarz 2021). The vascular endothelium is covered by a glycocalyx layer composed of glycoproteins and proteoglycans that form a molecular interface between circulating biomolecules and the endothelial surface (Kang et al. 2025). Under physiological conditions, VEGF is essential for embryonic development, wound healing, and tissue repair (Arsic et al. 2004; Matsumoto and Ema 2014). As the central regulator of blood vessel growth, VEGF is a critical target in regenerative medicine (Gianni-Barrera et al. 2020; Uccelli et al. 2019). Its ability to induce new vessel formation (angiogenesis) and enhance tissue recovery makes it particularly valuable in therapeutic applications aimed at restoring blood flow and repairing damaged tissues (Bao et al. 2009; Shams et al. 2022). Consequently, VEGF signaling has been widely explored in various therapeutic and diagnostic strategies, including nanomaterial-based biomedical platforms (Hao et al. 2024). Therefore, given its broad utility in both basic research and clinical applications, the efficient production and purification of high-purity VEGF is very important.

Currently, VEGF purification is achieved primarily through chromatography-based techniques, such as His-tag affinity chromatography, heparin affinity chromatography, and size exclusion chromatography (Zhou et al. 2021; Grosso et al. 2023; Ceylan et al. 2024). While these methods are effective in purifying high-purity VEGF, they have several limitations. These methods are time-consuming because they involve multistep processes and have restricted throughput due to flow rate limitations and high operational costs from expensive resins and equipment (Sánchez-Trasviña et al. 2021; Völzke et al. 2023). Moreover, column packing, maintenance, and optimization require specialized expertise and labor-intensive processes, further increasing the complexity and cost of production (Silva et al. 2025). Therefore, these challenges underscore the need for the development of alternative purification strategies that are simpler, faster, and more cost-effective, while maintaining high yield and purity.

**Fig. 1:**
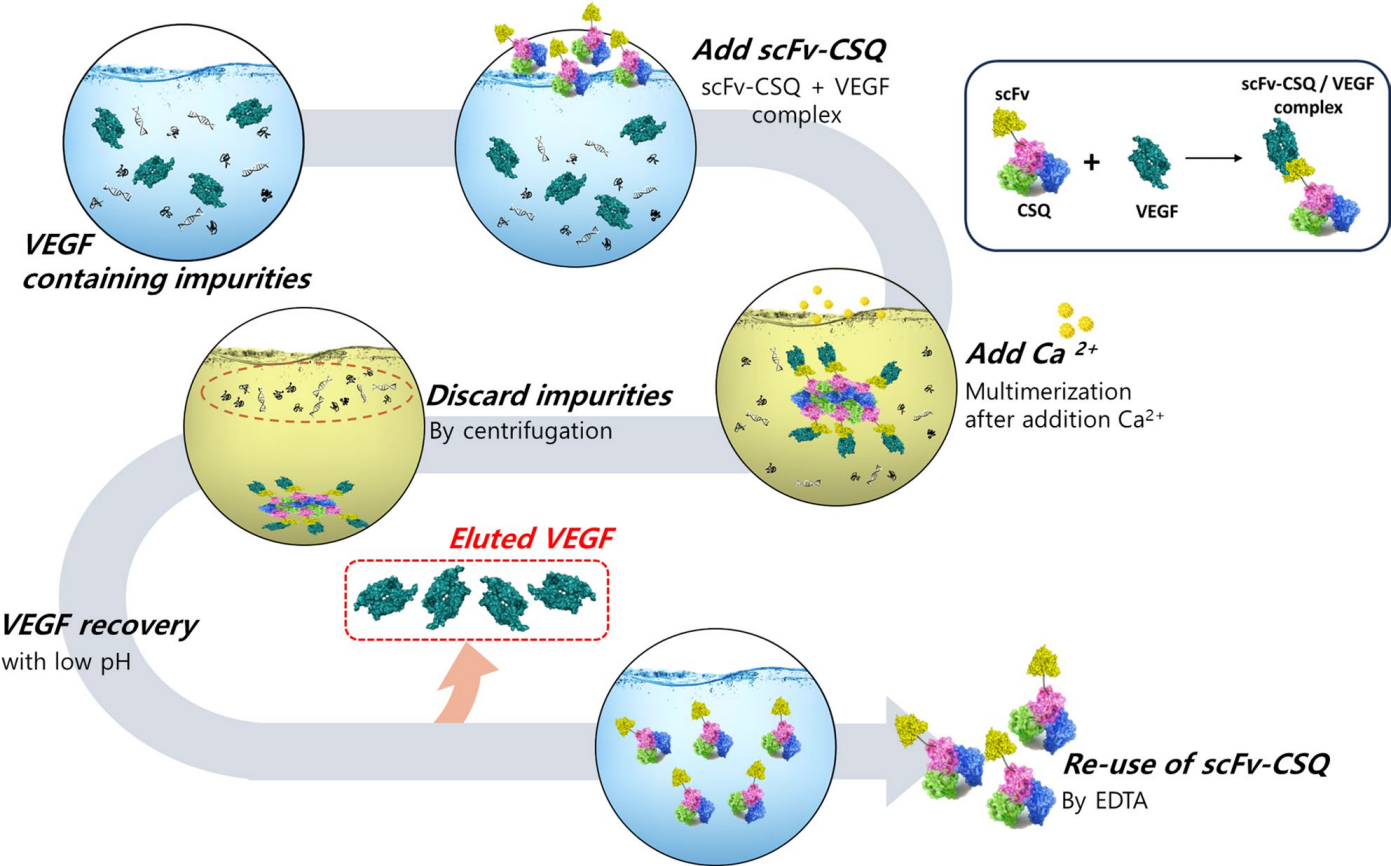
Schematic illustration of calcium-responsive purification of VEGF using anti-VEGF scFv-CSQ fusion protein. A diagram depicting the non-chromatographic affinity precipitation strategy for vascular endothelial growth factor (VEGF) purification. The engineered fusion protein, comprising an anti-VEGF single-chain variable fragment (scFv) and calsequestrin (CSQ), selectively binds to VEGF via the scFv domain. Upon Ca^2^⁺ addition, the CSQ domain undergoes calcium-induced polymerization, leading to phase transition and formation of VEGF-bound aggregates. Centrifugation enables easy separation of these aggregates from impurities. Finally, purified VEGF is dissociated and recovered under low pH conditions. This method provides a rapid, efficient, and resin-free alternative to conventional chromatography-based approaches

In this study, we present a novel non-chromatographic purification platform that enables simple, rapid and highly efficient isolation of VEGF using a calcium-responsive fusion protein composed of calsequestrin (CSQ) and an anti-VEGF single-chain variable fragment (scFv). This platform leverages the distinct biochemical properties of each component. The anti-VEGF scFv domain ensures the selective capture of VEGF molecules (Han et al. 2023; Sánchez-Trasviña et al. 2021), whereas the CSQ domain facilitates calcium-dependent polymerization, enabling efficient phase separation. Calsequestrin (CSQ) is a highly acidic calcium-binding protein found in muscle cells. Under low Ca^2^⁺ concentrations, CSQ exists as soluble monomers or dimers, but transitions into multimeric polymers as Ca^2^⁺ levels increase (Woo et al. 2020; Sibbles et al. 2022; Marabelli et al. 2023). By engineering a fusion protein that combines CSQ with anti-VEGF-scFv, we harnessed this calcium-dependent phase transition to drive the selective aggregation of VEGF-bound complexes. These aggregates can be readily separated from unbound impurities via low-speed centrifugation. The overall purification process, as illustrated in Fig. 1, consists of three streamlined steps: (i) specific capture of VEGF by the anti-VEGF-scFv-CSQ fusion protein, (ii) Ca^2^⁺ induced aggregation and removal of impurities, and (iii) recovery of purified VEGF through dissociation under low pH conditions.

This platform eliminates the need for conventional chromatography resins, complex instruments, and labor-intensive multiple workflows. By addressing the key limitations of traditional chromatography-based methods, it provides an innovative alternative that enables the rapid and cost-effective production of high-purity VEGF suitable for both research and therapeutic applications.

## Results

### Construction of the anti-VEGF-scFv-CSQ fusion protein

The anti-VEGF scFv-CSQ fusion protein was rationally engineered to enable efficient VEGF purification. The fusion protein was designed with the anti-VEGF scFv at the N-terminus, linked through a flexible peptide linker to the N-terminus of human cardiac calsequestrin (CSQ). This configuration preserves the VEGF-binding activity of the scFv domain and enables calcium-dependent polymerization via the CSQ domain for phase separation.

**Fig. 2:**
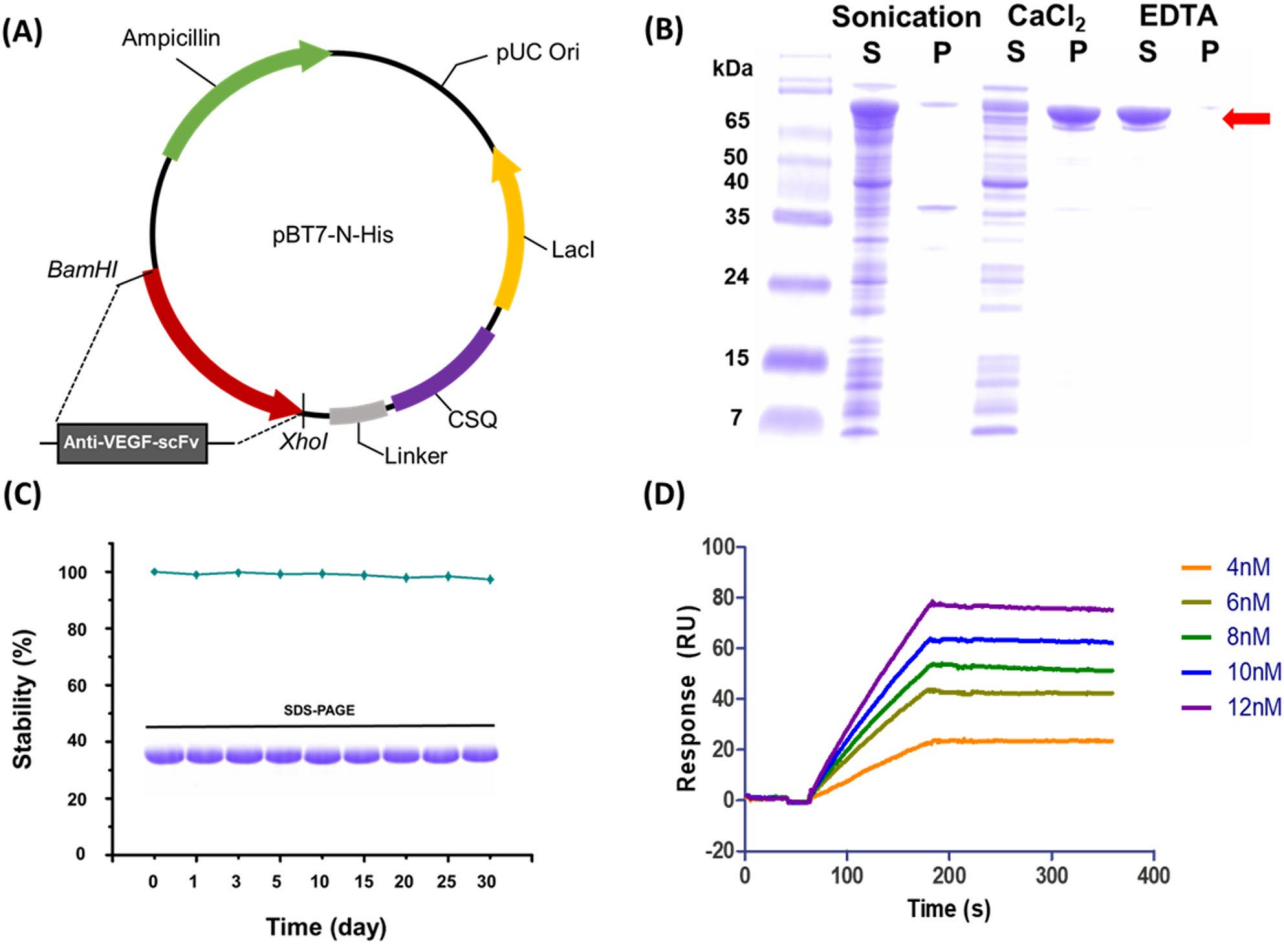
Production and functional analysis of the anti-VEGF-scFv-CSQ. **A** Schematic diagram of the anti-VEGF-scFv-CSQ protein structure. **B** SDS-PAGE analysis of proteins at each stage of anti-VEGF-scFv-CSQ purification by calcium-dependent phase transition. (S, supernatant; P, precipitate); **C** Long-term stability of anti-VEGF-scFv-CSQ at 4 °C. **D** Binding affinity (*K*_d_) of anti-VEGF-scFv-CSQ to VEGF, determined using surface plasmon resonance (SPR)

Anti-VEGF-scFv, comprising the heavy chain variable domain (VH) and light chain variable domain (VL), was selected as the VEGF affinity bait. Anti-VEGF-scFv maintains a high affinity for VEGF while lacking the Fc domain, which minimizes non-specific interactions. To reduce the functional interference between CSQ and anti-VEGF-scFv, the two proteins were fused using a flexible linker (GSEGSEGEGGSEGSEGEG) (Fig. 2A). The anti-VEGF-scFv-CSQ fusion protein was predominantly expressed in the soluble fraction of *Escherichia coli BL21*(DE3) (Supplementary Fig. 1). The obtained anti-VEGF-scFv-CSQ fusion protein was purified by calcium-dependent phase transition. At a critical concentration of CaCl₂ (10 mM), anti-VEGF-scFv-CSQ formed precipitates that were separated from the impurities. After centrifugation, the soluble impurities were easily removed. The precipitated anti-VEGF-scFv-CSQ was resolubilized by adding EDTA, yielding pure monomeric anti-VEGF-scFv-CSQ. Anti-VEGF-scFv-CSQ demonstrated over 96% solubility and was produced at 95% purity, as confirmed by SDS-PAGE (Fig. 2B).

Next, we assessed the long-term stability of anti-VEGF-scFv-CSQ at 4 °C. Over the 30-day incubation period, the quantity of anti-VEGF-scFv-CSQ decreased by less than 4%, demonstrating that anti-VEGF-scFv-CSQ possesses sufficient stability for use as a novel purification tool, and that this structural stability may contribute to the preservation of its functional properties (Fig. 2C). The CSQ protein can influence the structural and functional properties of anti-VEGF-scFv through conformational changes or steric hindrance. Therefore, the binding affinity between the anti-VEGF-scFv-CSQ and VEGF was evaluated using surface plasmon resonance (SPR)-based measurements. The dissociation constant (*K*_d_) was determined to be 1.1 nM, confirming that it has sufficient binding strength for VEGF purification (Fig. 2D). While this affinity is lower than that of high-affinity therapeutic antibodies such as brolucizumab (*K*_d_ = 28.4 pM), it remains sufficiently strong for effective VEGF capture and purification applications. These results suggest that fusion with CSQ does not critically impair the functional binding capacity of the scFv domain.

### Characterization of the calcium-dependent polymerization properties of anti-VEGF-scFv-CSQ fusion proteins

**Fig. 3:**
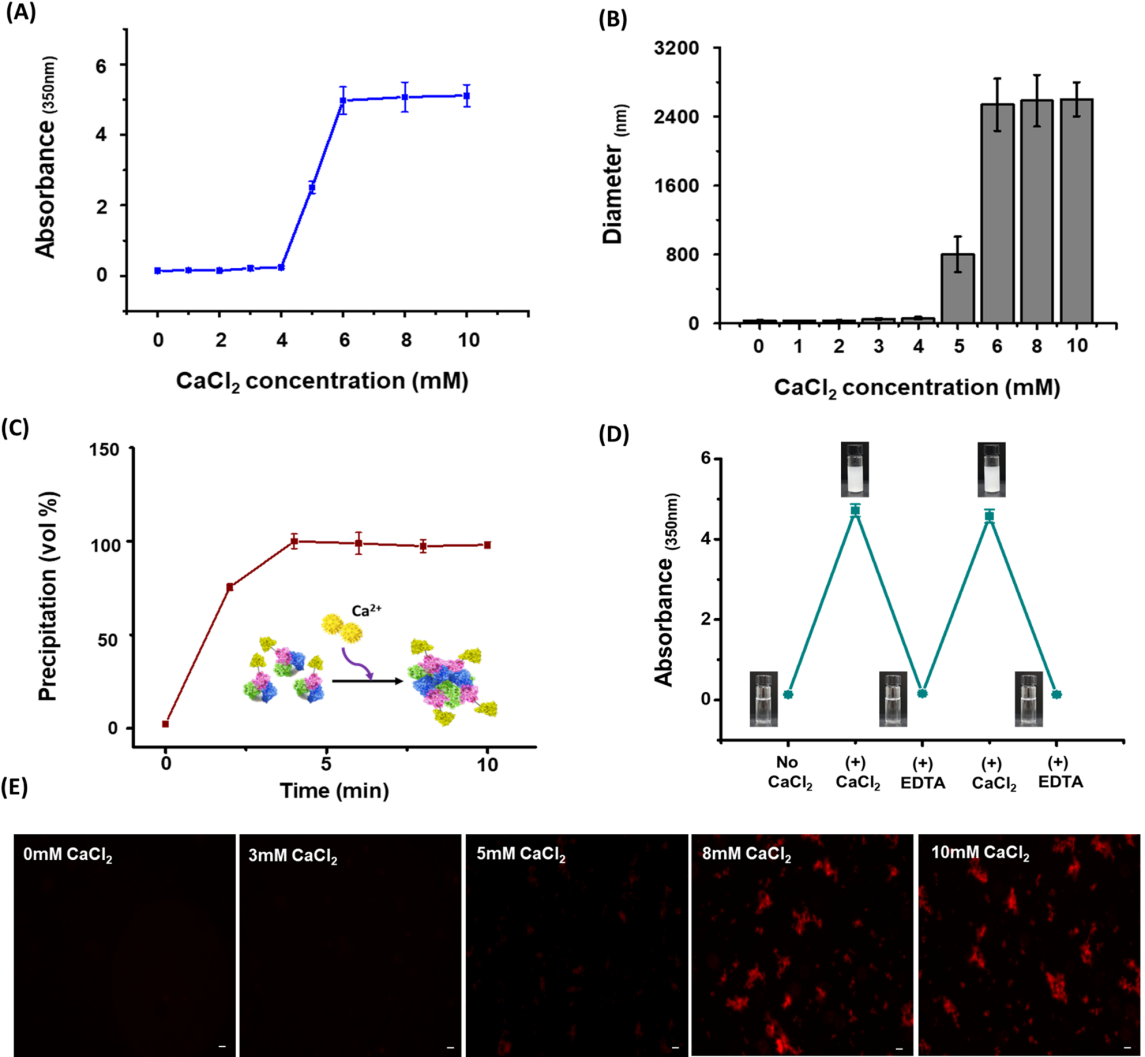
Characterization of anti-VEGF-scFv-CSQ Polymerization Properties. **A** Turbidity measurements of anti-VEGF-scFv-CSQ at 350 nm, showing the effect of the Ca^2^⁺ concentration. Data are presented as mean ± SD (n = 3). **B** Hydrodynamic size distribution of anti-VEGF-scFv-CSQ at increasing Ca^2^⁺ concentrations, measured using dynamic light scattering (DLS). Data are presented as mean ± SD (n = 5). **C** Polymerization kinetics of anti-VEGF-scFv-CSQ monitored over time with 10 mM CaCl₂. **D** Cyclical polymerization and depolymerization of anti-VEGF-scFv-CSQ induced by alternating addition of CaCl₂ and EDTA. Repeated cycles of CaCl₂ and EDTA did not affect the polymerization properties of the anti-VEGF-scFv-CSQ. **E** Fluorescence imaging of 5-TAMRA-labeled anti-VEGF-scFv-CSQ particles at various Ca.^2^⁺ concentrations (0, 3, 5, 8, and 10 mM CaCl₂)

Through characterization of the calcium-dependent polymerization and transition cycles of purified anti-VEGF-scFv-CSQ, we aimed to demonstrate the dual functionality of anti-VEGF-scFv-CSQ. Turbidity measurements are widely recognized as an effective approach for monitoring protein polymerization. As the concentration of Ca^2^⁺ increased, anti-VEGF-scFv-CSQ exhibited a significant increase in turbidity owing to protein polymerization, starting at 5 mM CaCl₂. The turbidity increased from 2.51 at 3 mM CaCl₂ to 4.97 at 6 mM CaCl₂, reaching saturation at CaCl₂ concentrations above 6 mM (Fig. 3A). Hydrodynamic size measurements using dynamic light scattering (DLS) similarly demonstrated this calcium-dependent polymerization behavior, with the particle size increasing sharply from 36 nm in the absence of CaCl₂ to 800 ± 200 nm at 5 mM CaCl₂ and further to 2580 ± 250 nm at concentrations above 6 mM CaCl₂ (Fig. 3B). The polymerization and aggregation of anti-VEGF-scFv-CSQ occurred rapidly, reaching saturation within 5 min after the addition of 10 mM CaCl₂ (Fig. 3C). Polymerization quantification showed that approximately 97.5% of anti-VEGF-scFv-CSQ was precipitated at 10 mM CaCl₂ (Supplementary Table 1). Furthermore, anti-VEGF-scFv-CSQ demonstrated a reversible phase transition, as the aggregates were completely dissolved upon EDTA treatment, which removed Ca^2^⁺ (Fig. 3D). To visualize the polymer morphology and calcium-dependent phase transition of anti-VEGF-scFv-CSQ, the protein was conjugated with 5-carboxy-tetramethylrhodamine (TAMRA) N-succinimidyl ester. The aggregation of anti-VEGF-scFv-CSQ was further visualized at Ca^2^⁺ concentrations of 0, 3, 5, 8, and 10 mM, revealing micrometer-sized aggregates consistent with the DLS data (Fig. 3E). These findings suggest that CSQ fused to anti-VEGF-scFv retains calcium-dependent properties, demonstrating the dual functionality of anti-VEGF-scFv-CSQ.

### Anti-VEGF-scFv-CSQ based affinity precipitation for VEGF

**Fig. 4:**
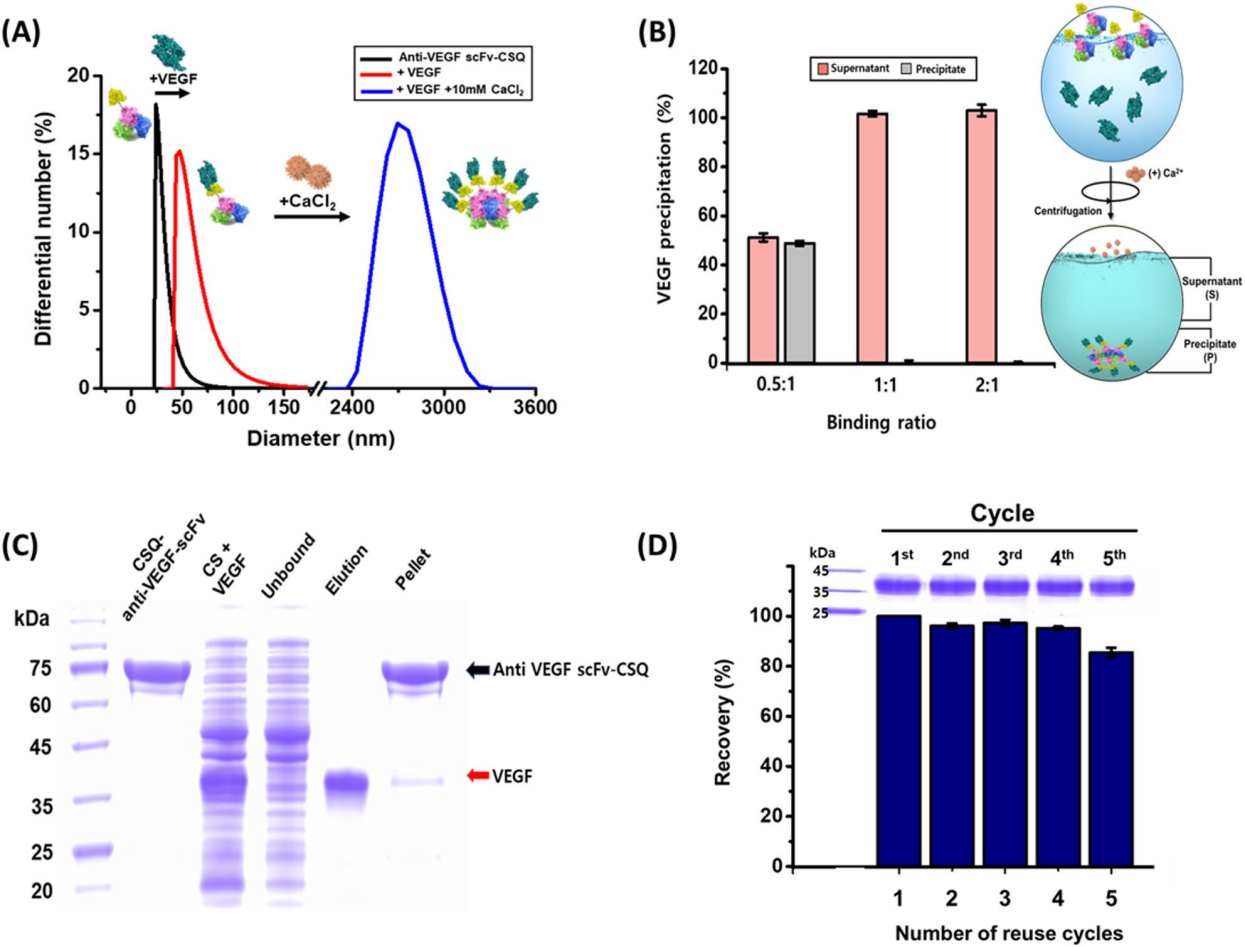
Analysis of VEGF Purification Using Anti-VEGF-scFv-CSQ. **A** Hydrodynamic size distribution of anti-VEGF-scFv-CSQ, the anti-VEGF-scFv-CSQ-VEGF complex, and polymerized anti-VEGF-scFv-CSQ-VEGF. **B** Binding ratio of anti-VEGF-scFv-CSQ to VEGF (S: supernatant, P: precipitate). **C** Purification of VEGF using Anti-VEGF-scFv-CSQ, analyzed by SDS-PAGE. **D** Reusability of anti-VEGF-scFv-CSQ for VEGF purification

To demonstrate that the anti-VEGF-scFv-CSQ fusion protein retained its Ca^2^⁺-dependent aggregation properties and VEGF binding ability even when bound to VEGF, hydrodynamic size measurements were performed under various conditions. Anti-VEGF-scFv-CSQ showed a slight increase in size to 45 nm upon binding to VEGF, whereas a dramatic increase in size to approximately 2700 nm was observed in the presence of 10 mM CaCl₂. These results indicate that scFv (28 kDa) does not interfere with the Ca^2^⁺ responsiveness or VEGF binding ability of the fusion protein (Fig. 4A). To purify VEGF, the binding conditions between anti-VEGF-scFv and VEGF were optimized. Considering the monovalent nature of the scFv and the dimeric structure of VEGF, a 1:1 molar ratio was used as a practical condition, while 0.5:1 and 2:1 ratios were tested to evaluate binding under sub-stoichiometric and excess conditions. The proteins were incubated for 10 min, followed by the addition of 10 mM CaCl₂. The resulting precipitates were analyzed using SDS-PAGE to determine the optimal binding ratio of anti-VEGF-scFv to VEGF. At molar ratios of 1:1 and 2:1, nearly complete precipitation of VEGF was observed (Fig. 4B, Supplementary Fig. 2).

After the optimal experimental conditions were established, VEGF purification was performed and evaluated. VEGF-containing impurities were incubated with anti-VEGF-scFv-CSQ at 4 °C for 5 min to allow binding. Subsequently, 10 mM CaCl₂ was added to induce aggregation of the anti-VEGF-scFv-CSQ-VEGF complex for 5 min, followed by centrifugation at 2800 × g for 5 min to remove impurities. The captured VEGF was separated from the complex by incubating the precipitate in 50 mM glycine buffer (pH 2.5) for 10 min. SDS-PAGE analysis showed that highly pure VEGF (94%) was recovered with a yield of 93% within 30 min of the entire process (Fig. 4C). Consistently, SEC–HPLC analysis demonstrated a purity of 94.3% for the purified VEGF (Supplementary Fig. 3A). We also measured the concentration of contaminating host cell proteins (HCPs). The HCP content dramatically decreased from 1.44 × 10^4^ ppm to 774 ppm after purification (Supplementary Fig. 3B).

When five repeated rounds of VEGF purification were performed using a fixed amount of anti-VEGF-scFv-CSQ, SDS-PAGE analysis revealed that the overall recovery yield of VEGF was consistently maintained above ~ 85% (Fig. 4D).

## Discussion

This study introduces a novel non-chromatographic purification method for VEGF that leverages the calcium-dependent polymerization properties of the anti-VEGF-scFv-CSQ fusion protein. Vascular endothelial growth factor (VEGF) is a key regulator of angiogenesis and is widely used in regenerative medicine and therapeutic research, which has created increasing demand for efficient production and purification strategies. Currently, the purification of recombinant VEGF largely depends on affinity chromatography, which requires costly chromatographic columns, specialized equipment, and multiple processing steps. In addition, chromatographic capture is often influenced by diffusion-limited mass transfer within porous resins, which can reduce binding efficiency and limit overall process throughput, further increasing operational complexity and cost, particularly in large-scale production (Tallarek et al. 1999; Niezen et al. 2024). By utilizing the calcium-responsive behavior of calsequestrin (CSQ), this platform successfully overcomes several limitations associated with traditional chromatography-based techniques, including high cost and complex workflows.

Notably, anti-VEGF-scFv-CSQ retained a high binding affinity for VEGF and exhibited rapid and reversible Ca^2^⁺-dependent polymerization, enabling efficient separation of VEGF from impurities. This column-free mechanism reduces operational costs and technical complexity, making the platform cost-effective and scalable for industrial applications. The purification process achieved high purity (94%) and yield (93%) within 30 min, suggesting a more rapid purification process than conventional affinity resin-based methods. Anti-VEGF-scFv-CSQ maintained stable purification performance over five repeated cycles. Furthermore, the feasibility of extending this platform to other target proteins was supported by anti-CD3-scFv-CSQ, which retained calcium-dependent polymerization and binding activity (Supplementary Fig. 4). These results suggest that this strategy could enable efficient purification of diverse scFv targets.

This strategy also offers advantages in terms of process scalability. Because the separation step is driven by bulk-phase aggregation rather than column flow dynamics, the process can potentially be scaled using simple solid–liquid separation methods such as centrifugation. Furthermore, integration with scalable separation technologies, such as tangential flow filtration (TFF), may allow efficient process scale-up through size-based separation of the polymerized complexes.

Despite these results, several limitations should be considered. First, further investigations addressing potential endotoxin contamination, immunogenicity, and additional polishing steps may be necessary before clinical use. Second, while the current study demonstrates the feasibility and efficiency of the purification strategy at the laboratory scale, further optimization of process conditions and operational parameters will be required to fully assess its performance under industrial-scale production settings. By addressing these challenges, this platform has the potential to enable practical applications in protein purification and biomaterials.

## Materials and methods

### Construction of the anti-VEGF-scFv-CSQ fusion protein

For the construction of the anti-VEGF-scFv-CSQ fusion protein, a pBT7-N-His vector containing the human cardiac calsequestrin (CSQ; amino acids 20–399) sequence was synthesized by Bioneer (Daejeon, Korea). The gene encoding the anti-VEGF single-chain variable fragment (scFv) was synthesized by Bioneer (Daejeon, Korea). A flexible linker sequence (GSEGSEGEGGSEGSEGEG) was designed and incorporated between the scFv and CSQ coding regions to minimize potential steric interference. The scFv insert was cloned into the CSQ-containing pBT7-N-His vector using *BamHI* and *XhoI* restriction sites, and the final construct was confirmed by DNA sequencing (Bioneer, Daejeon, Korea). The anti-VEGF-scFv clone was transformed into chemically competent *Escherichia coli* strain BL21 (DE3) (Enzynomics, Daejeon, Korea).

### Expression and purification of anti-VEGF-scFv-CSQ

For the expression of anti-VEGF-scFv-CSQ protein, expression stocks were cultured in 5 mL of terrific broth combined with 10 mg/mL ampicillin (Sigma) for 4 h. The pre-culture was then inoculated into 250 mL of fresh medium. The 250 mL culture was incubated at 37 °C for 2 h with shaking at 150 rpm until the optical density at 600 nm (OD_600_) reached 0.8–1. Protein expression was induced by adding 250 µL of 1 M isopropyl β-D-1-thiogalactopyranoside (IPTG; LPS solution) to the flask, followed by incubation at 20 °C with shaking at 150 rpm for 16 h. After incubation, the cells were pelleted and resuspended in 20 mM Tris buffer. The cells were lysed by pulse sonication on ice (130 W, 20 kHz, pulse cycle of 2 s on and 2 s off for at least 2 min until complete cell disruption), and the lysate was treated with 20% phenylmethylsulfonyl fluoride (PMSF) to inactivate proteases. The lysate was centrifuged at 18,000 × g for 1 h at 4 °C to separate the soluble fraction. Anti-VEGF-scFv-CSQ was purified from the soluble fraction using a calcium-dependent phase transition. Polymerization was induced by adding CaCl₂ to a final concentration of 20 mM, followed by incubation at 4 °C for 30 min. This step facilitated the selective aggregation of the CSQ-fused proteins, allowing them to be separated from other impurities. Aggregated proteins were pelleted by centrifugation at 2800 × g for 20 min at 4 °C. The resulting pellet was resuspended in 20 mM Tris buffer containing 40 mM EDTA to depolymerize the protein complexes. The sample was subjected to buffer exchange using a HiPrep 26/10 Desalting column (Cytiva, formerly GE Healthcare) on an ÄKTA go FPLC system (Cytiva, formerly GE Healthcare). Purified anti-VEGF-scFv-CSQ was stored at 4 °C for up to 30 days to assess its long-term stability. All purified proteins were analyzed by sodium dodecyl sulfate–polyacrylamide gel electrophoresis (SDS-PAGE) on 15% polyacrylamide gels.

### Binding affinity analysis

The binding affinity of anti-VEGF-scFv for VEGF was assessed using surface plasmon resonance (SPR) on a BIACORE 3000 system (GE Healthcare, Little Chalfont, UK). Anti-VEGF-scFv-CSQ solutions at concentrations of 4, 6, 8, 10, and 12 nM were flowed over the VEGF-coated sensor surface at a rate of 30 μL/min. Each concentration was injected in an association phase lasting 2 min, followed by a dissociation phase of 10 min in PBS (pH 7.4). The interaction kinetics were analyzed using the BIA evaluation 2.1 software (GE Healthcare, Little Chalfont, UK).

### Anti-VEGF-scFv to VEGF binding ratio

For the binding ratio experiments, 10 µM samples of VEGF were mixed with 5–20 µM anti-VEGF-scFv (0.5:1 to 2:1) in 20 mM Tris (pH 7.0). CaCl₂ (10 mM) was mixed at room temperature before centrifugation at 2800 × g for 30 min at 4 °C. The resulting supernatants and pellets were analyzed using SDS-PAGE.

### VEGF purification using anti-VEGF-scFv-CSQ

Anti-VEGF-scFv 500µL (10 µM) was mixed with VEGF 500µL (20 µM) containing impurities and incubated at 4 °C for 5 min. To the anti-VEGF-scFv-VEGF complex, 10 mM CaCl₂ was added to ensure robust precipitation, and the mixture was incubated at 4 °C for 5 min to induce the formation of insoluble precipitates. The sample was centrifuged at 2,800 × g for 5 min at 4 °C, and the supernatant was discarded. The insoluble pellet was resuspended in 100 µL of 20 mM Tris + 40 mM EDTA (pH 9.0) to dissociate the calcium-induced aggregates. Subsequently, 900 µL of 50 mM glycine buffer (pH 2.5) was immediately added to elute VEGF from the anti-VEGF-scFv-CSQ complex. The mixture was incubated at room temperature for 10 min to facilitate VEGF elution. Upon centrifugation, the supernatant containing purified VEGF was collected and immediately neutralized with 1 M Tris buffer (pH 9.0). Under acidic conditions, the CSQ-based fusion protein re-precipitated and remained in the pellet. Purified VEGF samples were analyzed by SDS-PAGE. To evaluate reusability, five consecutive purification cycles were performed using fresh VEGF in each round. After each VEGF recovery, the retained anti-VEGF-scFv-CSQ pellet was reused under identical conditions.

## Supplementary Information

Below is the link to the electronic supplementary material.

Supplementary Material 1.

## Data Availability

All data generated or analysed during this study are included in this published article and its supplementary information files.

## References

[CR1] Arsic N, Zacchigna S, Zentilin L, Ramirez-Correa G, Pattarini L, Salvi A, Sinagra G, Giacca M (2004) Vascular endothelial growth factor stimulates skeletal muscle regeneration in vivo. Mol Ther 10:844–854 15509502 10.1016/j.ymthe.2004.08.007

[CR2] Bao P, Kodra A, Tomic-Canic M, Golinko MS, Ehrlich HP, Brem H (2009) The role of vascular endothelial growth factor in wound healing. J Surg Res 153:347–358 19027922 10.1016/j.jss.2008.04.023 PMC2728016

[CR3] Brown L, Detmar M, Claffey K, Nagy J, Feng D, Dvorak A, Dvorak H (1997) Vascular permeability factor/vascular endothelial growth factor: a multifunctional angiogenic cytokine. In: Regulation of angiogenesis. pp 233–269 10.1007/978-3-0348-9006-9_10 9002222

[CR4] Ceylan HK et al (2024) A colorimetric immunoassay for the detection of human vascular endothelial growth factor 165 (VEGF165) based on anti-VEGF iron oxide nanoparticle conjugation. Microchim Acta 191:133 10.1007/s00604-024-06228-0 PMC10867064 38353782

[CR5] Gianni-Barrera R, Di Maggio N, Melly L, Burger MG, Mujagic E, Gürke L, Schaefer DJ, Banfi A (2020) Therapeutic vascularization in regenerative medicine. Stem Cells Transl Med 9:433–444 31922362 10.1002/sctm.19-0319 PMC7103618

[CR6] Grosso A et al (2023) VEGF dose controls the coupling of angiogenesis and osteogenesis in engineered bone. Npj Regen Med 8:15 36914692 10.1038/s41536-023-00288-1 PMC10011536

[CR7] Han N, Xu X, Liu Y, Luo G (2023) AAV2-antiVEGFscFv gene therapy for retinal neovascularization. Mol Ther Methods Clin Dev 31 10.1016/j.omtm.2023.101145 PMC10679950 38027065

[CR8] Hao X et al (2024) Nanomaterials for bone metastasis. J Control Release 373:640–651 39084467 10.1016/j.jconrel.2024.07.067

[CR9] Kang H et al (2025) The substructure of the endothelial glycocalyx in rat aorta and its interaction with low-density lipoproteins (LDL). Am J Pathol 10.1016/j.ajpath.2025.06.005 PMC12597546 40645580

[CR10] Marabelli C, Santiago DJ, Priori SG (2023) The structural–functional crosstalk of the calsequestrin system: insights and pathological implications. Biomolecules 13:1693 38136565 10.3390/biom13121693 PMC10741413

[CR11] Matsumoto K, Ema M (2014) Roles of VEGF-A signalling in development, regeneration, and tumours. J Biochem 156:1–10 24839295 10.1093/jb/mvu031

[CR12] Niezen LE et al (2024) Detailed analysis of the effective and intra-particle diffusion coefficient of proteins at elevated pressure in columns packed with wide-pore core-shell particles. J Chromatogr A 1713:464538 38043163 10.1016/j.chroma.2023.464538

[CR13] Sánchez-Trasviña C, Flores-Gatica M, Enriquez-Ochoa D, Rito-Palomares M, Mayolo-Deloisa K (2021) Purification of modified therapeutic proteins available on the market: an analysis of chromatography-based strategies. Front Bioeng Biotechnol 9:717326 34490225 10.3389/fbioe.2021.717326 PMC8417561

[CR14] Shams F, Moravvej H, Hosseinzadeh S, Mostafavi E, Bayat H, Kazemi B, Bandehpour M, Rostami E, Rahimpour A, Moosavian H (2022) Overexpression of VEGF in dermal fibroblast cells accelerates angiogenesis and wound healing: in vitro and in vivo studies. Sci Rep 12:18529 36323953 10.1038/s41598-022-23304-8 PMC9630276

[CR15] Sibbles ET, Waddell HM, Mereacre V, Jones PP, Munro ML (2022) The function and regulation of calsequestrin-2: implications in calcium-mediated arrhythmias. Biophys Rev. 10.1007/s12551-021-00914-6 35340602 10.1007/s12551-021-00914-6 PMC8921388

[CR16] Silva TC et al (2025) Optimization of multi-column chromatography for capture and polishing at high protein load. Biotechnol Prog 41:e70047 40485304 10.1002/btpr.70047 PMC12531920

[CR17] Tallarek U, Vergeldt FJ, Van As H (1999) Stagnant mobile phase mass transfer in chromatographic media: intraparticle diffusion and exchange kinetics. J Phys Chem B 103:7654–7664

[CR18] Uccelli A, Wolff T, Valente P, Di Maggio N, Pellegrino M, Gürke L, Banfi A, Gianni-Barrera R (2019) Vascular endothelial growth factor biology for regenerative angiogenesis. Swiss Med Wkly 149:w20011 30685867 10.4414/smw.2019.20011

[CR19] Völzke JL, Smatty S, Döring S, Ewald S, Oelze M, Fratzke F, Flemig S, Konthur Z, Weller MG (2023) Efficient purification of polyhistidine-tagged recombinant proteins using functionalized corundum particles. Biotech 12:31 37218748 10.3390/biotech12020031 PMC10204482

[CR20] Wiszniak S, Schwarz Q (2021) Exploring the intracrine functions of VEGF-A. Biomolecules 11:128 33478167 10.3390/biom11010128 PMC7835749

[CR21] Woo JS, Jeong SY, Park JH, Choi JH, Lee EH (2020) Calsequestrin: a well-known but curious protein in skeletal muscle. Exp Mol Med 52:1908–1925 33288873 10.1038/s12276-020-00535-1 PMC8080761

[CR22] Zhou W et al (2021) Production of high-purity recombinant human vascular endothelial growth factor (rhVEGF165) by Pichia pastoris. Chin J Biotechnol 37:4083–4094 10.13345/j.cjb.210021 34841808

