# Supplementary material for: A simple, rapid, resin-free platform for purification of vascular endothelial growth factor using a calcium-responsive fusion protein
Source: Bioresour Bioprocess. 2026 May 4;13(1):63. doi: 10.1186/s40643-026-01063-y (PMC13139529; doi:10.1186/s40643-026-01063-y)
Supplement: Supplementary file 1 — Supplementary Material 1. [file 40643_2026_1063_MOESM1_ESM.docx]

**Supplementary Data**

**Supplementary methods**

**Calcium-Dependent Turbidity and Polymerization Kinetics**

Anti-VEGF-scFv-CSQ polymerization was induced by incubating the protein with various concentrations (0, 1, 2, 3, 4, 5, 6, 8, 10 mM) of CaCl₂ for 30 minutes at 4 ℃. For anti-CD3-scFv-CSQ, polymerization was evaluated under the same conditions using CaCl₂ concentrations of 0, 2, 4, 6, 8, and 10 mM. Turbidity was determined by measuring the absorbance at 350 nm as a function of CaCl₂ concentration using a UV-Vis spectrophotometer (NanoDrop One, Thermo Fisher Scientific, Waltham, USA). To evaluate the time-dependent precipitation behavior of anti-VEGF-scFv-CSQ, 10 mM CaCl₂ was added to the purified protein and incubated at 4 ℃. Aliquots were collected at 0, 2, 4, 6, 8, and 10 minutes, and immediately centrifuged at 2,800 × g for 10 minutes at 4 ℃ to separate the soluble and precipitated fractions. The supernatants were analyzed by SDS-PAGE on 15% gels, and the remaining soluble protein was quantified by densitometric analysis using a FUSION FX6 EDGE imaging system (Vilber Lourmat, France).

**Calcium-Dependent Inverse-Transition Cycle**

For the inverse-transition cycle, 10 mM CaCl₂ was added to anti-VEGF-scFv-CSQ, and the absorbance at 350 nm was measured. Subsequently, 20 mM EDTA was added to the sample, the mixture was vortexed, and the absorbance at 350 nm was measured again. The same sample was then treated with 20 mM CaCl₂ and the measurement cycle was repeated.

**Stability of anti-VEGF-scFv-CSQ**

Purified anti-VEGF-scFv-CSQ (20 μM) was incubated at 4 ℃ for up to 30 days. Aliquots were collected on days 1, 3, 5, 10, 15, 20, and 25, and the remaining protein quantity was analyzed by SDS-PAGE. Band intensity was quantified using Image J software to evaluate changes in relative protein quantity over time. All measurements were performed in triplicate.

**Morphology analysis: Fluorescence microscopy**

Anti-VEGF-scFv-CSQ (1 mg/mL) in PBS buffer (pH 7.4) was conjugated with a 10-fold molar excess of 5-carboxy-tetramethylrhodamine (TAMRA) N-succinimidyl ester (Sigma) at room temperature for 1 hour under continuous mixing. TAMRA-labeled anti-VEGF-scFv-CSQ was separated from the free TAMRA dye using a desalting column (GE Healthcare, Piscataway, NJ, USA). TAMRA-labeled anti-VEGF-scFv-CSQ samples were then prepared in a buffer containing 20 mM Tris (pH 7.0) with varying concentrations of CaCl₂ (0, 3, 5, 8, or 10 mM) and incubated at 4 ℃ for 30 minutes. After incubation, fluorescent signals were visualized using a fluorescence microscope (DMI 3000B; Leica, Wetzlar, Germany). Images were captured using a × 63 objective lens.

**Hydrodynamic Size Distribution and Sequential Protein Binding and Phase-Transition Behavior Analysis**

The hydrodynamic size distribution of the anti-VEGF-scFv-CSQ complexes was measured at room temperature using dynamic light scattering (DLS) with a Zetasizer Nano Range (ELSZ-2000, Otsuka). The analysis was conducted on anti-VEGF-scFv-CSQ protein (1 mg/mL) in the presence or absence of varying concentrations of CaCl_2_, ranging from 1 to 10 mM (1, 2, 3, 5, 6, 8, and 10). Anti-CD3-scFv-CSQ was also analyzed under the same conditions using CaCl_2_ concentrations of 0, 2, 4, 6, 8, and 10 mM. To further evaluate ligand-binding and calcium-induced phase transition the hydrodynamic size distributions of the anti-VEGF-scFv-CSQ-VEGF complexes and polymerized anti-VEGF-scFv-CSQ-VEGF were evaluated using DLS under the same conditions. anti-VEGF-scFv-CSQ protein (10 µM) was incubated with or without 10 µM VEGF for 30 minutes prior to analysis. To induce polymerization, CaCl_2_ was added to the anti-VEGF-scFv-CSQ-VEGF complexes, and size measurements were performed using DLS at room temperature.

**SEC HPLC**

The purity of anti-VEGF-scFv was evaluated by size-exclusion high-performance liquid chromatography (SEC–HPLC) using a YL9100 Plus system (Anyang, Korea) with an SEC column (YMC, Kyoto, Japan). Samples (20 µL) were injected for analysis at a flow rate of 0.2 mL/min.

**Quantification of Host Cell Protein Contamination**

HCP contaminants were quantified using a Host Cell Protein ELISA detection kit (Canopy Biosciences, MI, USA) according to the manufacturer’s instructions. To evaluate the purification efficiency, VEGF samples collected before and after purification were analyzed. The final HCP levels were expressed in parts per million (ppm).

**ELISA for anti-CD3-scFv binding kinetics**

High-binding 96-well plates (Corning, USA) were coated with human CD3ε/δ heterodimer (50 μg/mL; Acro Biosystems, USA). After coating, the plates were blocked with 2% BSA and washed with PBS. anti-CD3-scFv-CSQ samples were added to the wells and incubated. Following washes with PBST, bound scFv was detected using a mouse monoclonal anti-hexahistidine antibody (1:3,000; Proteintech, USA; Cat. No. HRP-66005), followed by incubation with an HRP-conjugated goat anti-mouse secondary antibody (1:5,000; Santa Cruz Biotechnology, USA; Cat. No. sc-2354). After

washing, TMB substrate was added to develop the signal, and the reaction was terminated before measuring absorbance at 450 nm.

**
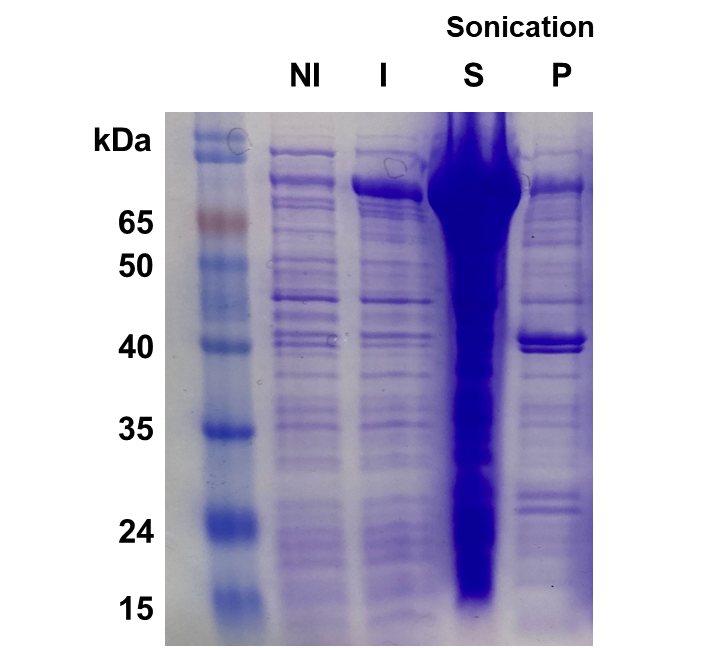
**

**Supplementary Figure 1. SDS–PAGE analysis of anti-VEGF-scFv-CSQ expression. (**NI: not induction; I: induction; S: soluble fraction; P: insoluble fraction.)


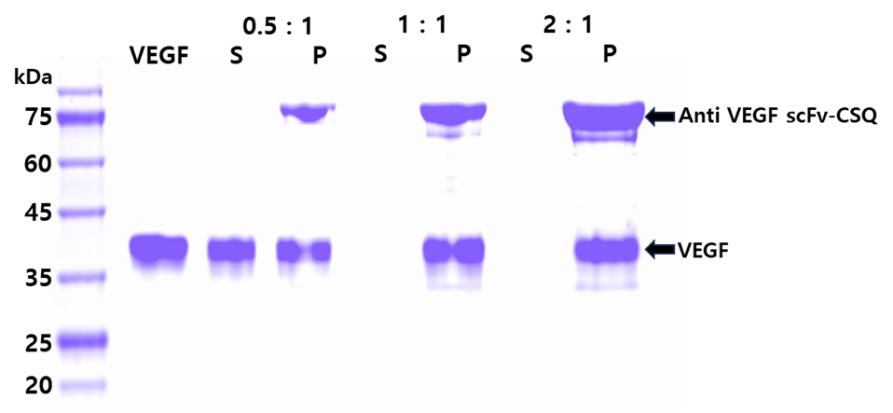


**Supplementary Figure 2. SDS-PAGE analysis of the binding ratio of anti-VEGF-scFv-CSQ to VEGF.** (S: supernatant containing unbound VEGF, P: calcium-dependent precipitate containing bound VEGF)

**
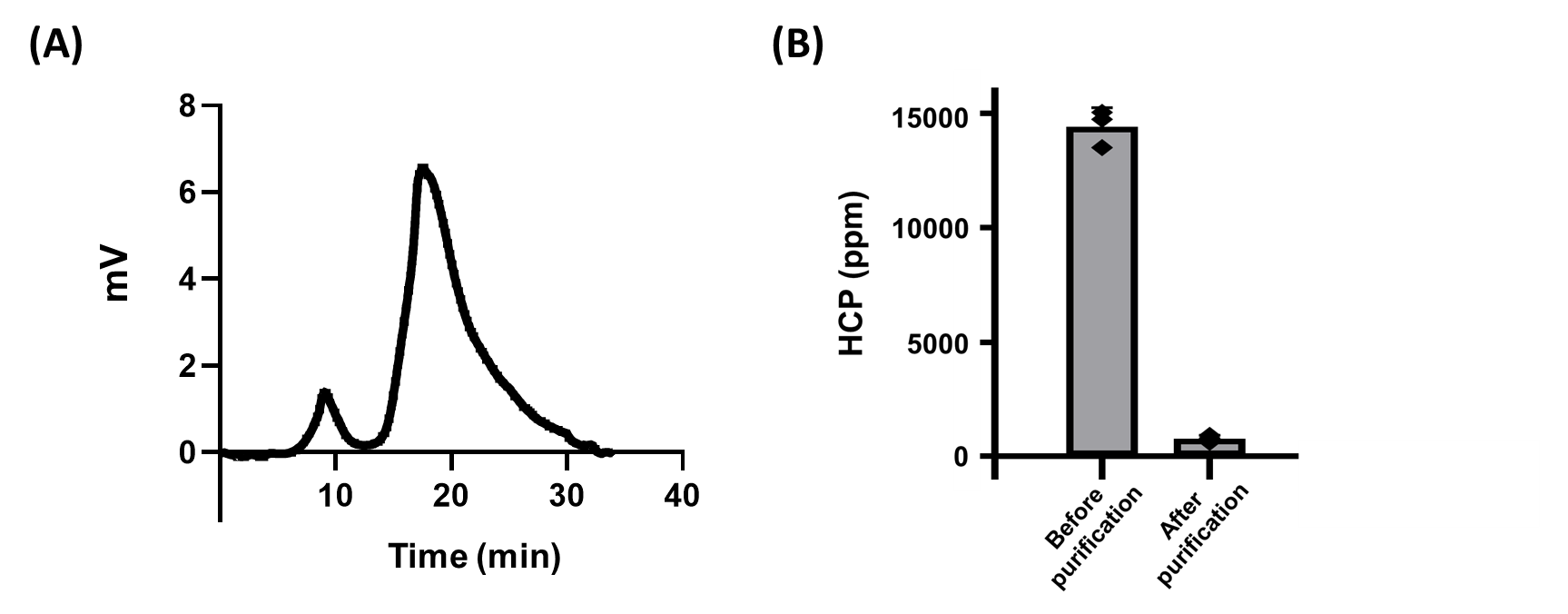
**

**Supplementary Figure 3. Purity analysis of VEGF.** (A) SEC–HPLC analysis of purified VEGF. (B) Quantification of host cell protein (HCP) contamination in VEGF samples before and after purification.


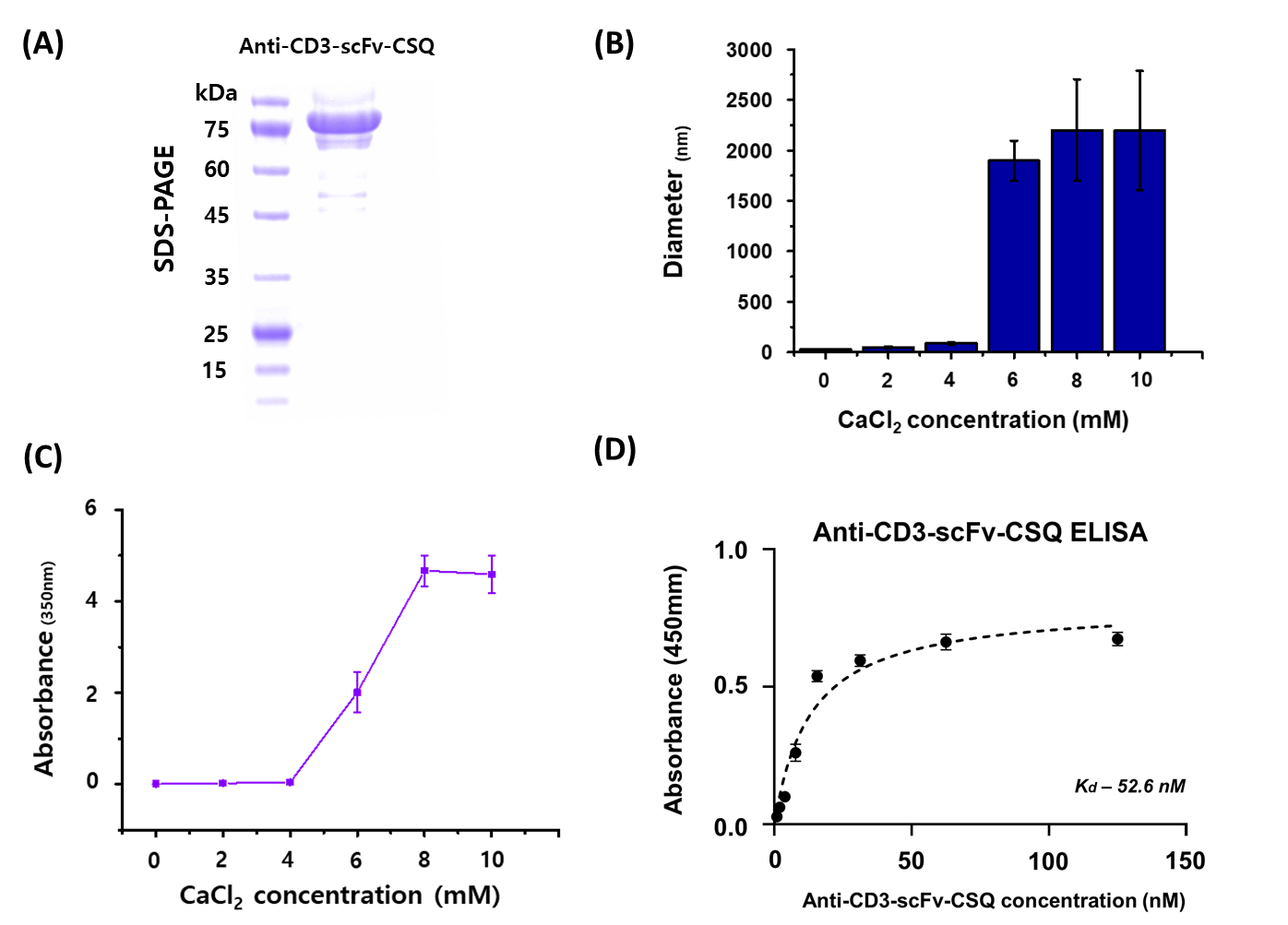


**Supplementary Figure 4. Evaluation of the applicability of the CSQ platform using anti-CD3-scFv-CSQ.** (A) SDS-PAGE analysis of anti-CD3-scFv-CSQ. (B) Turbidity of anti-CD3-scFv-CSQ by Ca²⁺ was measured at 350 nm. (C) Hydrodynamic size distribution of anti-CD3-scFv-CSQ at increasing Ca²⁺ concentrations, measured using dynamic light scattering (DLS). (D) ELISA binding of anti-CD3-scFv-CSQ. Data are presented as mean ± SD (n = 3).

| **Ca²⁺ (mM)** | **Supernatant protein (mg/mL)** | **Polymerized fraction (%)** |
| --- | --- | --- |
| **0** | 2.00 | 0.0 |
| **1** | 1.94 | 3.0 |
| **2** | 1.95 | 2.5 |
| **3** | 1.86 | 7.0 |
| **4** | 1.83 | 8.5 |
| **5** | 0.96 | 52.0 |
| **6** | 0.14 | 93.0 |
| **8** | 0.03 | 98.5 |
| **10** | 0.02 | 99.0 |

**Supplementary Table 1. Quantification of calcium-dependent polymerization of anti-VEGF-scFv-CSQ.** Remaining soluble protein in the supernatant after Ca²⁺-dependent polymerization of 2 mg/ml anti-VEGF-scFv-CSQ was quantified using a BCA assay.
